# Rapid Progression of a T3-T4 Paraspinal Schwannoma in a Young Female

**DOI:** 10.7759/cureus.62773

**Published:** 2024-06-20

**Authors:** Riya Rupareliya, Monica Khadka, Devaun M Reid, Scott Taylor

**Affiliations:** 1 Medicine, Florida State University College of Medicine, Tallahassee, USA; 2 Internal Medicine, University of South Florida Morsani College of Medicine, Tampa, USA; 3 Pathology, Ketchum, Wood, and Burgert (KWB) Pathology Associates, Tallahassee, USA

**Keywords:** rapid-growing tumor, tinel test, peripheral nerve sheath tumors, neuropathic pain, paraspinal schwannoma

## Abstract

This case report presents an unusual incidence of a T3-T4 paraspinal schwannoma in a 22-year-old female, highlighting its clinical significance due to its atypical presentation and growth rate. Schwannomas, benign peripheral nerve sheath tumors, are typically slow-growing and present with minimal or no neuropathic symptoms. However, this case deviated from the norm, with the patient experiencing significant neuropathic pain and rapid tumor growth from 37 mm to 55 mm over a period of six months, necessitating surgical intervention. Unique to this case was the presence of a positive Tinel sign and localized neuropathic back pain, features not commonly associated with paraspinal schwannomas. Through MRI and histological evaluation, the diagnosis of schwannoma was confirmed, underlining the necessity of considering paraspinal schwannomas in differential diagnoses for patients presenting similar symptoms. This case contributes to the medical literature by emphasizing the variability in presentation and growth rates of schwannomas, reinforcing the need for a thorough evaluation and an individualized approach to management in young patients presenting with neuropathic pain and positive neurological signs.

## Introduction

Schwannomas are benign tumors originating from Schwann cells, which form protective sheaths around peripheral nerves [1]. Although these tumors are generally slow-growing and often present minimal symptoms, they can occur anywhere in the body, sometimes leading to significant clinical challenges, particularly when they exhibit atypical characteristics [1,2]. They most commonly occur between the second and fifth decades of life but can occur at any age [3]. This study illustrates the implications of such atypical schwannomas, emphasizing the necessity for medical professionals to consider these tumors in the differential diagnosis of young patients with unexpected neuropathic symptoms or rapid growth. This case highlights the importance of a comprehensive diagnostic approach and tailored management strategies for affected individuals. Through detailed magnetic resonance imaging (MRI) and histological evaluations, this discussion offers valuable insights into diagnosing and managing schwannomas, enhancing our understanding of their clinical spectrum.

## Case presentation

We present the case of a 22-year-old female with a past medical history of mild costochondritis who presented to an orthopedic clinic with complaints of electric, shock-like pain in the upper left quadrant of her back for the past four months. There was no history of trauma or any reports of constitutional symptoms such as weight loss or fever. Previous X-ray scans showed no significant bony abnormalities. On examination, there was a 2 cm raised area in the T3-T4 area that was slowly growing. There was tenderness to palpation and a positive Tinel sign (nerve pain elicited by tapping the raised area) to the skin overlying the posterior shoulder and left upper back covering dermatomes C8-T3. There were no signs of muscle wasting or weakness. A neurological examination of the upper and lower limbs was normal, including normal strength testing and intact reflexes. MRI of the thoracic spine with and without intravenous contrast showed a 3.7 cm height, 1.7 cm diameter, and 1.3 cm width ovoid circumscribed soft-tissue mass in the left posterior paramedian upper back in the T3-T4 paraspinal musculature. There was no adjacent thoracic vertebral body bone marrow edema or spinal canal stenosis. The MRI findings were in favor of a peripheral nerve sheath tumor, likely a schwannoma and a follow-up ultrasound-guided biopsy was recommended. Other differential diagnoses included a giant cell tumor.

**Figure 1:**
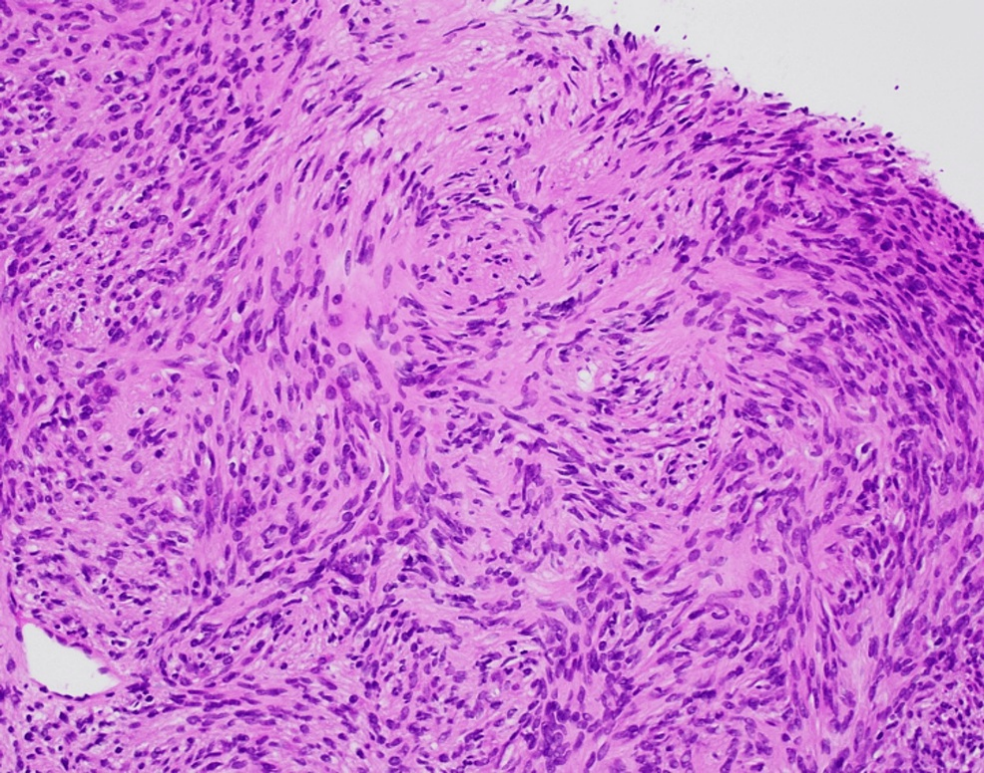
A high-power view demonstrating alternating areas of hyper- and hypocellularity of bland spindled cells with thin, wavy, and elongated nuclei with tapered ends. The image is original and was produced by the authors.

**Figure 2:**
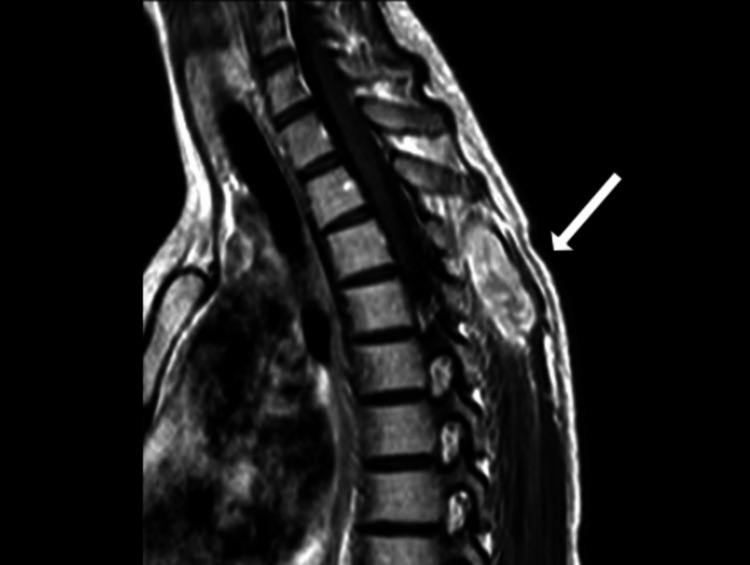
Sagittal T2-weighted MRI image finding of the mass that had grown to 4.1 x 1.7 x 1.5 cm in height, diameter, and width, respectively. The white arrow points to the mass.

A microscopic examination of the initial ultrasound-guided biopsy showed a spindle lesion with the presence of both hypercellular and hypocellular (Antoni A and Antoni B, respectively) areas (Figure 1). The tumor cells were positive for S-100 and SOX-10 and negative for Melan A and Pancytokeratin. Pathological findings supported the diagnosis of schwannoma. An additional MRI was taken four months after the initial diagnosis, and the findings showed that the mass had grown to 4.1 x 1.7 x 1.5 cm in height, diameter, and width, respectively (Figure 2).

**Figure 3:**
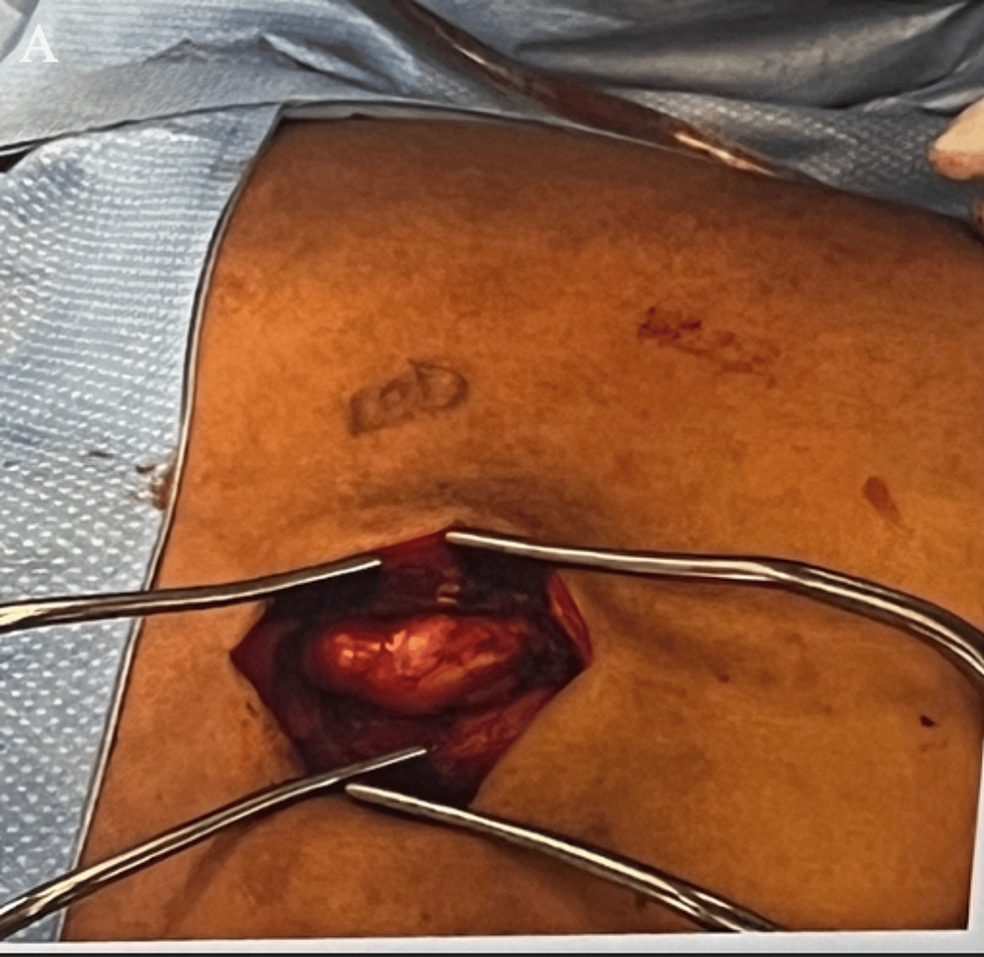
Intraoperative image of the tumor in the fascial plane between the multifidus and longissimus heads of the erector spinae muscle. The image is original and was produced by the authors.

**Figure 4:**
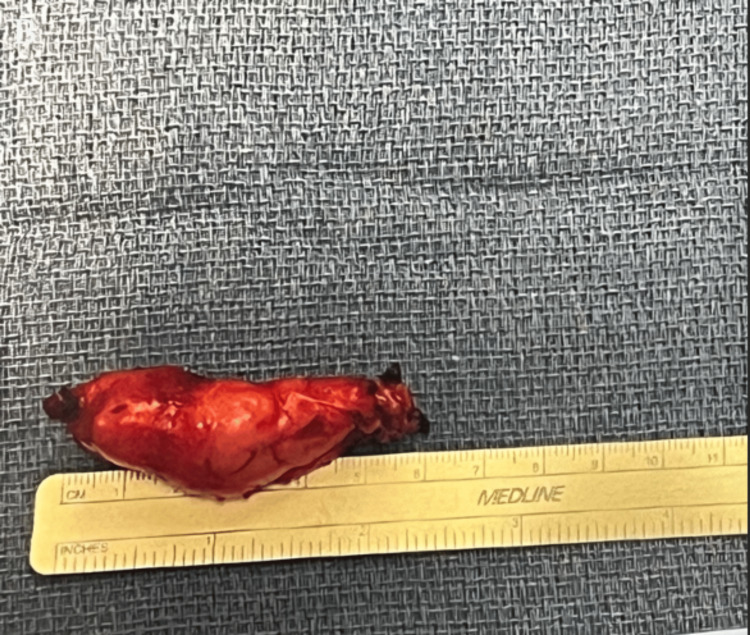
The gross image of the tumor fully resected. The image is original and was produced by the authors.

Given the rapid growth of the lesion and the increasing neuropathic pain experienced throughout this period, complete surgical removal was recommended. Surgical excision of the schwannoma was done six months later. An excision was made down to the deep fascia overlying paraspinal muscles on the left upper back, and the schwannoma was found in the fascial plane between the multifidus and longissimus heads of the erector spinae muscle (Figure 3). The schwannoma was further infiltrating down to the lamina of the associated vertebra, but there was no entry into the spinal canal. The mass was resected in its entirety and was measured to be 5.5 x 1.5 x 1.4 cm (Figure 4). No originating nerve was identified. The patient reports loss of cutaneous sensation to the skin overlying the incision area three months postoperatively. This deficit is likely due to the transection of a cutaneous nerve when resecting the tumor. Neuropathic pain has been resolved entirely.

## Discussion

Solitary soft-tissue tumors are generally rare and challenging to diagnose, particularly in younger demographics [4]. When considering soft-tissue masses, the differential diagnosis includes lipoma (16%), fibrous histiocytoma (13%), nodular fasciitis (11%), hemangioma (8%), fibromatosis (7%), neurofibroma (5%), and schwannoma (5%) [5]. Schwannomas are benign peripheral nerve sheath tumors arising from Schwann cells and can occur anywhere in the body, with approximately 25%-45% occurring in the head and neck region [6]. The presence of an intramuscular schwannoma is a rare occurrence [7]. The origin of a schwannoma found in the paraspinal T3-T4 musculature is unknown but likely arises from the dorsal rami of the thoracic nerves. There have been few previous reports of paraspinal schwannomas arising from the dorsal ramus nerve in the lumbar and dorsal spinal areas in patients between the third and fourth decades of life. One such case discussed the occurrence of a paraspinal schwannoma originating from the dorsal ramus nerve in a 62-year-old located between the erector spinae muscle and the L2-L4 region [8]. Another discusses a similar occurrence, but in a 45-year-old male patient with a mass located in the erector spinae muscles D9-D11, presenting with no pain [9]. Because paraspinal schwannomas tend to originate from small motor nerve branches, neurological symptoms, including pain, motor weakness, Tinel sign, and paresthesia, are rare [7-11].

In the previous three cases of reported paraspinal schwannomas, no neuropathic pain was reported by the patients, and none had a positive Tinel sign [8,9,12]. The presenting case is unique in that the paraspinal schwannoma was found in a patient who was in her second decade of life and who reported experiencing considerable neuropathic pain radiating to her left arm due to a mass effect on nearby cutaneous nerves. The physical examination showed tenderness to palpation and a positive Tinel sign, further reinforcing the uniqueness of the case. Before surgical intervention, a trial of muscle relaxants, nonsteroidal anti-inflammatory drugs (NSAIDs), acetaminophen, and gabapentin was used but did not provide effective pain management.

Though paraspinal intramuscular schwannomas can be clinically different from non-paraspinal intramuscular schwannomas, they are generally slow-growing. On average, schwannomas grow 1-3 mm annually [13]. In our present case, however, the tumor size at diagnosis in February was 37 mm in height and was found to be 55 mm when entirely excised. This amounted to a total growth of 18 mm, suggesting that the growth rate of intramuscular schwannomas may differ from that of non-intramuscular schwannomas. The final histopathologic findings on the excisional specimen were consistent with the previous biopsy pathologic findings and strongly supported the diagnosis of a schwannoma. Normal histological findings of a schwannoma include the presence of alternating hypercellularity (Antoni A) and hypocellularity (Antoni B), as well as diffuse, strong expression of S100 [14].

## Conclusions

This case of a T3-T4 paraspinal schwannoma found in a 22-year-old female patient offers unique insights. The schwannoma in our current case demonstrates an unusual presentation of neuropathic symptoms and further displays an atypical growth rate, necessitating total resection. This case suggests that a paraspinal schwannoma should be considered in the differential diagnosis for a patient presenting with localized neuropathic back pain and the presence of a positive Tinel sign. MRI and histological evaluation can further help narrow down the diagnosis to a schwannoma.
